# Primary CD34^+^ cells of patients with vacuoles, E1 enzyme, X‐linked, autoinflammatory, somatic (VEXAS) syndrome are highly sensitive to targeted treatment with TAK‐243

**DOI:** 10.1111/bjh.70203

**Published:** 2025-10-12

**Authors:** Daniel Nowak, Marie Demmerle, Alessa Klär, Alexander Streuer, Vladimir Riabov, Eva Altrock, Felicitas Rapp, Ann‐Christin Belzer, Teresa Klink, Meret Hahn, Franziska Hofmann, Verena Nowak, Nadine Weimer, Julia Obländer, Iris Palme, Melda Göl, Ali Darwich, Laurenz Steiner, Mohammed Abba, Georgia Metzgeroth, Wolf‐Karsten Hofmann, Nanni Schmitt

**Affiliations:** ^1^ Department of Hematology and Oncology, Medical Faculty Mannheim Heidelberg University Mannheim Germany; ^2^ Department of Orthopedic Surgery, Medical Faculty Mannheim Heidelberg University Mannheim Germany

**Keywords:** MDS, pevonedistat, TAK‐243, VEXAS syndrome

To the Editor,

Vacuoles, E1 enzyme, X‐linked, autoinflammatory, somatic (VEXAS) syndrome is a systemic autoinflammatory disease characterized by somatically acquired inactivating mutations of the *UBA1* gene in haematopoietic stem and progenitor cells.^1^ *UBA1* encodes the ubiquitin‐like modifier‐activating enzyme 1, which plays a crucial role in the post‐translational modification processes of ubiquitination and atypical neddylation responsible for the regulation of protein degradation and cellular homeostasis.^2^, ^3^ VEXAS syndrome predominantly affects men in late adulthood, typically after the age of 50, and is characterized by severe, treatment refractory inflammation, including recurrent fever, chondritis, neutrophilic dermatosis, alveolitis, vasculitis and cytopenias. Additionally, VEXAS patients have a predisposition for the development of myeloid malignancies such as myelodysplastic neoplasms (MDS).^4^ Morphologically, VEXAS presents with hypercellular bone marrow, varying degrees of dysplasia, macrocytic anaemia and, most prominently, vacuolated myeloid and erythroid precursors.^5^

Given the recent discovery of VEXAS syndrome, standardized treatment options are not yet established, leaving patients with high morbidity and mortality.^6^ Disease management primarily involves anti‐inflammatory therapies such as high‐dose glucocorticoids, which are associated with major toxicity.^7^ Treatment with the hypomethylating agent azacitidine and Janus kinase (JAK) inhibitors achieved mixed results, while high incidences of severe infections were found to be frequent adverse events.^8^, ^9^, ^10^ The only potentially curative approach as of today is an allogeneic stem cell transplantation.

Complete loss of UBA1 function is non‐viable to cells and organisms.^11^ The most common mutations in VEXAS syndrome are *UBA1 p.M41T*, *UBA1 p.M41V* and *UBA1 p.M41L*, which affect methionine 41 of exon 3 and lead to isoforms of UBA1 with considerably reduced catalytic activity.^12^ However, current scientific data suggest that residual catalytic activity is retained in cells of VEXAS, which enables affected myeloid progenitors to survive and gain a selective clonal advantage.^1^ Therefore, a particularly promising approach for molecularly targeted treatment of VEXAS syndrome is the use of drugs that act on the ubiquitin proteasome system. Two potential candidates are the inhibitors TAK‐243 (MLN7243), which targets E1 ubiquitin‐activating enzymes (UAEs), and pevonedistat (TAK‐924, MLN4924), which targets NEDD8‐activating enzymes (NAEs).^13^, ^14^

Recent research by Chiaramida and colleagues demonstrated the therapeutic potential of TAK‐243 using a murine Uba1M41L knock‐in cell line model that replicated VEXAS syndrome features.^15^ Building on these findings, for this study, we obtained primary bone marrow samples from patients with VEXAS syndrome (*n* = 5) and MDS (*n* = 10) as well as healthy, age‐matched controls (*n* = 10) from femoral heads after hip replacement surgery, and conducted in vitro testing of TAK‐243 and pevonedistat on primary CD34^+^ cells to explore their clinical activity. Both the MDS and the healthy cohort consist of *n* = 5 samples of both sexes. The MDS samples (*n* = 5 MDS with low blasts (MDS‐LB), *n* = 5 MDS‐SF3B1) were exclusively selected from patients with normal karyotype, low blast counts and low risk according to Revised International Prognostic Scoring System (IPSS‐R). Patients with *DNMT3A* and *TET2* mutations were preferentially selected to match the co‐mutation profile of VEXAS patients. Detailed information on patients and controls is provided in Tables S1 and S2.

Examination of the clinical characteristics of the five VEXAS patients revealed several common features of VEXAS syndrome (Table S1). At the time of the bone marrow aspiration, the mean age was 72.4 years. All VEXAS patients presented with inflammatory symptoms, elevated C‐reactive protein levels and MDS as haematological comorbidity. Four of five VEXAS patients received prednisolone. Vacuolization of granulopoiesis and/or erythropoiesis was observed in all patients. All five VEXAS patients had missense variants of codon 41: two patients harboured the hot spot mutation *p.Met41Leu* (variant allele frequency [VAF] 83% and 82%), while the other three carried the *p.Met41Thr* variant (VAF 90%, 89% and 36%). Furthermore, panel sequencing revealed the VEXAS‐typical co‐mutation *DNMT3A* in two patients (VAF 40% and 15%). The presence of *UBA1* variants in CD34^+^ cells from VEXAS patients was confirmed using droplet digital polymerase chain reaction (ddPCR) (Figure S1A–E).

To assess the therapeutic potential of TAK‐243 and pevonedistat, VEXAS, MDS and healthy CD34^+^ cells were enriched from mononuclear cells using magnetic cell separation yielding a purity of at least 90%. CD34^+^ cells were cultured in StemSpan™ SFEM II (STEMCELL Technologies) plus cytokines (10 ng/mL fibroblast growth factor 1 (FGF‐1), 50 ng/mL FMS‐like tyrosine kinase 3 ligand (FLT3‐L), 50 ng/mL stem cell factor (SCF), 10 ng/mL thrombopoietin (TPO)) and 1% pen‐strep and treated with different concentrations of TAK‐243 (range: 0.24–1000 nM) and pevonedistat (range: 0.78–3000 nM) or vehicle (dimethyl sulfoxide (DMSO)) for 48 h. Cell viability and apoptosis were then assessed using the CellTiter‐Glo Luminescent Cell Viability Assay and Caspase‐Glo 3/7 Assay (Promega) respectively. All results were normalized according to the DMSO results. The methods used are described in more detail in the Supporting Information: Methods section.

Our findings revealed that primary VEXAS CD34^+^ cells exhibit significantly higher sensitivity to TAK‐243, with a half maximal inhibitory concentration (IC50) of 22 nM, compared to MDS (133 nM, *p* = 0.0047) and healthy cells (192 nM, *p* = 0.0005) as demonstrated by decreasing cell viability (Figure 1A,B). Furthermore, MDS cells were more susceptible to treatment with TAK‐243 than healthy CD34^+^ cells. Pevonedistat also showed increased cytotoxic effects in VEXAS cells (IC50 553 nM) compared to MDS (1048 nM, *p* = 0.0004) and healthy CD34^+^ cells (1107 nM, *p* = 0.0002) (Figure 1C,D). IC50 results for pevonedistat in MDS and healthy CD34^+^ cells were nearly identical. Overall, MDS and healthy samples only responded to higher concentrations of TAK‐243 (>100 nM) and pevonedistat (>500 nM). Notably, minimal variability was observed in the IC50 results for TAK‐243 and pevonedistat across all samples of each entity (Figures S2A–F and S5A–F).

**FIGURE 1:**
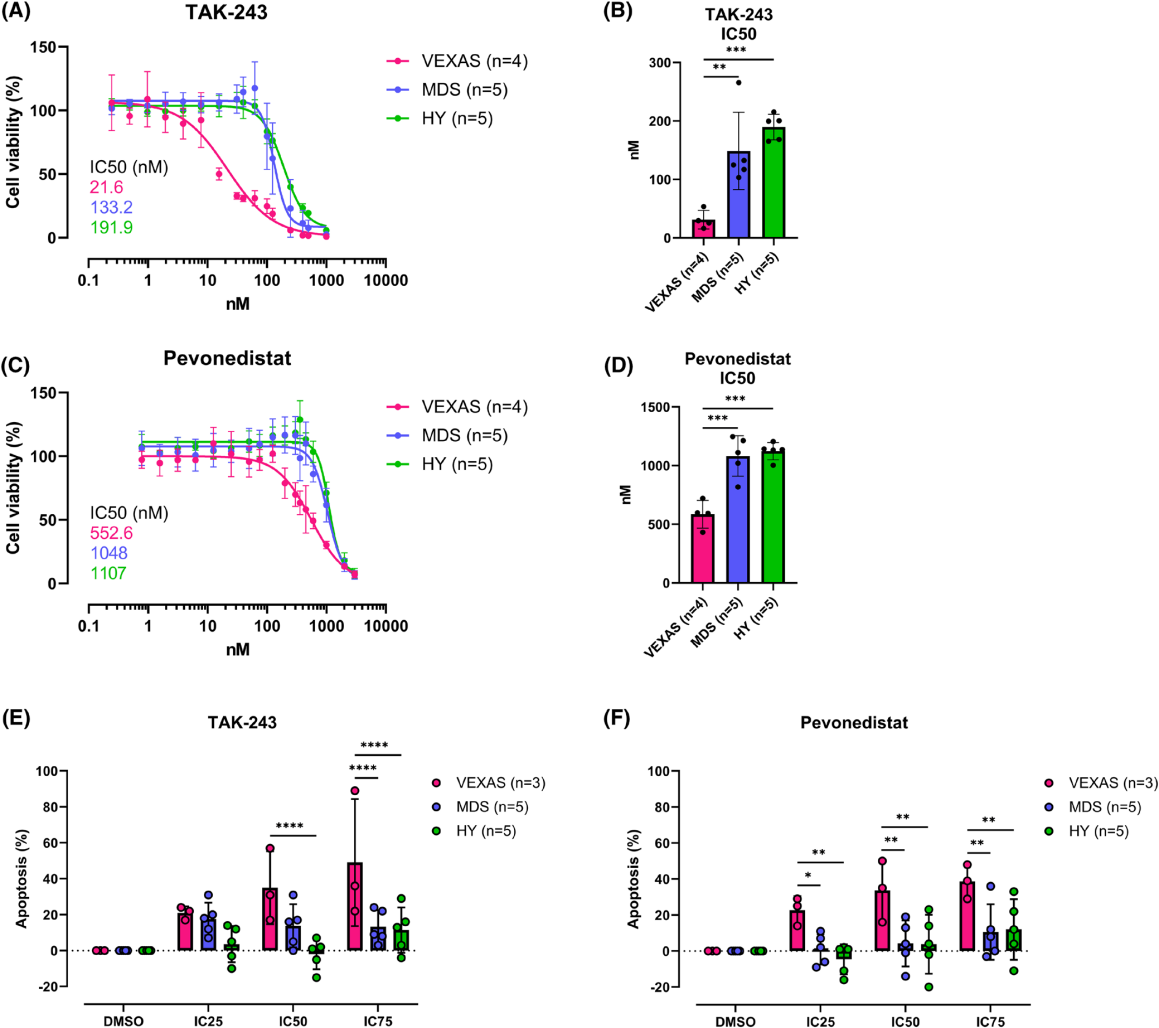
VEXAS CD34^+^ cells are highly sensitive to treatment with TAK‐243 and pevonedistat compared to MDS and healthy samples. Dose–response experiments of primary VEXAS (*n* = 3–4; magenta), MDS (*n* = 5; blue) and healthy (*n* = 5; green) CD34^+^ cells treated with TAK‐243 or pevonedistat. (A–D) Cell viability was measured 48 h after treatment using CellTiter‐Glo® luminescent cell viability assay. (A, C) Dose response curves of TAK‐243 (A) and pevonedistat (C). (B, D) IC50s of TAK‐243 (B) and pevonedistat (D). (E, F) The percentage of apoptotic cells was measured after 48 h using Caspase‐Glo® 3/7 Assay. The IC25, IC50 and IC75 values for TAK‐243 (E) and pevonedistat (F) for CD34^+^ cells of VEXAS patients, calculated based on the results from (A–D) were used for the treatment of all three entities. Data were analysed using nonlinear regression (A, C), two‐tailed unpaired *t*‐test (B, D) and ordinary two‐way analysis of variance (anova) (E, F) using Prism 10 (GraphPad Software), and are represented as mean ± SD. *p ≤ 0.05, **p ≤ 0.01, ***p ≤ 0.001, ****p ≤ 0.0001. MDS, myelodysplastic neoplasms; VEXAS, vacuoles, E1 enzyme, X‐linked, autoinflammatory, somatic.

Interestingly, CD34^+^ cells of all three groups demonstrated significantly higher sensitivity to TAK‐243 than to pevonedistat, as evidenced by lower IC50 values for cell viability (*p* < 0.0001 for all) (Figure S3A–F). This difference can most probably be attributed to the fact that pevonedistat, unlike TAK‐243, does not directly interfere with the ubiquitination process, but as an inhibitor of NAE affects neddylation. Although UBA1 is also involved in the process of atypical neddylation, mutations in *UBA1* likely have a weaker effect on this specific pathway.

Furthermore, we could demonstrate that VEXAS CD34^+^ cells exhibited significantly higher levels of apoptosis after treatment with TAK‐243 and pevonedistat compared to both MDS (TAK‐243: IC75 *p* = 0.0007; pevonedistat: IC25 *p* = 0.035, IC50 *p* = 0.0031, IC75 *p* = 0.0049) and healthy CD34^+^ cells (TAK‐243: IC50 *p* = 0.0005, IC75 *p* = 0.0004; pevonedistat: IC25 *p* = 0.0064, IC50 *p* = 0.0027, IC75 *p* = 0.0077) (Figure 1E,F). The differences between MDS and healthy CD34^+^ cells were again not significant for any of the concentrations. These results provide further evidence that TAK‐243 is more effective than pevonedistat in killing VEXAS CD34^+^ cells. Due to the high levels of apoptosis, it can be assumed that TAK‐243 and pevonedistat primarily induce apoptotic cell death, which is probably triggered by the disruption of the ubiquitin–proteasome pathway. In general, it should also be noted that even when analysing exclusively male MDS and healthy samples, the overall results of the viability and apoptosis assays remained similar (Figure S4A–F).

To investigate in detail whether TAK‐243 and pevonedistat primarily eliminate *UBA1*‐mutated CD34^+^ cells due to their mechanisms of action, we determined the *UBA1* VAF of the remaining cells after treatment. For this purpose, we performed additional experiments with larger cell numbers in order to isolate sufficient amounts of DNA. This required prior expansion of some of the CD34^+^ samples (see Supporting Information: Methods for details). Treatment with TAK‐243 and pevonedistat was carried out under the same conditions as in the previous experiments. The *UBA1* VAF was then determined using ddPCR. The results of these experiments showed that TAK‐243, but not pevonedistat, reduced the *UBA1* VAF of VEXAS CD34^+^ cells in a concentration‐dependent manner (Figure 2A,B; Table S3). In addition, based on the ddPCR results, we reconstructed the proportion of *UBA1*‐mutated and non‐mutated cells in the CD34^+^ samples (Figure 2C,D), which further emphasized the selective cytotoxic effect of TAK‐243. These findings, combined with the results of the viability and apoptosis assays, strongly indicate that TAK‐243 is indeed a targeted treatment for *UBA1*‐mutated cells.

**FIGURE 2:**
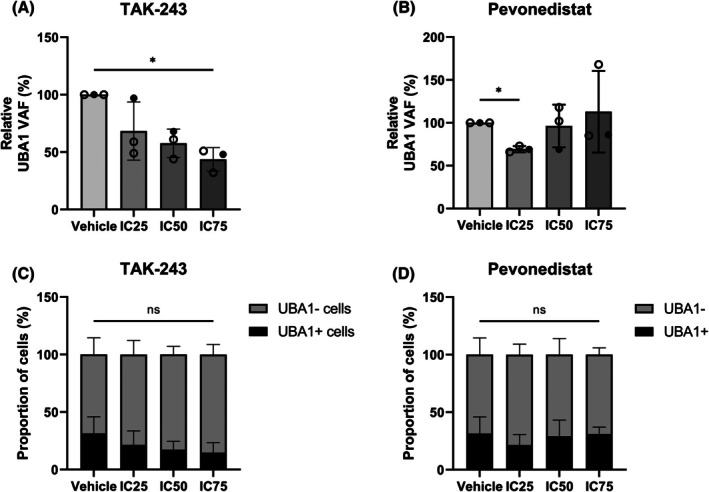
TAK‐243 selectively targets *UBA1*‐mutated VEXAS CD34^+^ cells. Non‐expanded (black dots) and expanded (circles) CD34^+^ cells of patients VEXAS1, VEXAS4 and VEXAS5 were treated with the IC25, IC50 and IC75 values of TAK‐243 (A) or pevonedistat (B) for 48 h. The cells were then harvested for DNA isolation. (A, B) The variant allele frequency (VAF) of *UBA1* for each condition was determined by droplet digital PCR (ddPCR). Vehicle control was set to 100% and the other values were normalized accordingly. (C, D) Based on the *UBA1* VAFs determined by ddPCR, the proportion of *UBA1*‐mutated and non‐mutated cells was determined. The raw data of the ddPCR results can be found in Table S3. Data were analysed using ordinary one‐way (A, B) or two‐way (C, D) analysis of variance (anova) using Prism 10 (GraphPad Software), and are represented as mean ± SD. ns, not significant, *p ≤ 0.05. VEXAS, vacuoles, E1 enzyme, X‐linked, autoinflammatory, somatic.

In the case of pevonedistat, a reduction in the *UBA1* VAF was only clearly observed at IC25. The values for IC50 and IC75 showed no change or even a tendency towards an increase in the *UBA1* VAF compared to the vehicle control. This may suggest that healthy, or at least the non‐mutated, haematopoiesis experiences similar or greater cytotoxicity from treatment with pevonedistat than *UBA1*‐mutated haematopoiesis. However, these findings contrast with those of the viability and apoptosis assays, in which the VEXAS CD34^+^ cells were significantly more sensitive to treatment with pevonedistat than cells from MDS patients and healthy controls. Ultimately, this could mean that the blockade of neddylation by pevonedistat is not sufficient to exploit the intrinsic vulnerability of *UBA1*‐mutated cells. After all, strong inhibition of this pathway inevitably leads to cell death even in healthy cells. Nevertheless, it should be noted as a limiting factor that the increased *UBA1* VAFs were only observed in the two expanded CD34^+^ samples. It remains unclear to what extent this cultivation step may have affected the VEXAS CD34^+^ cells and their response to the substances.

Overall, our study provides preclinical evidence supporting the potential of TAK‐243 as a molecularly targeted therapy for VEXAS syndrome. Primary CD34^+^ cells from VEXAS patients exhibited significantly higher sensitivity to both TAK‐243 and pevonedistat compared to cells from MDS patients and healthy controls as demonstrated by lower cell viabilities and higher levels of apoptosis. However, only TAK‐243 could reduce the proportion of *UBA1*‐mutated CD34^+^ cells in a concentration‐dependent manner. Taken together, these results could indicate a broad therapeutic window in which, hypothetically, malignant haematopoiesis could be eradicated but healthy haematopoiesis is not yet affected. Furthermore, our data suggest that treatment with TAK‐243 selectively targets *UBA1*‐mutated myeloid progenitor cells, which are the origin of the inflammatory disease, by inducing synthetic lethality while sparing healthy haematopoietic progenitors. This increased selective susceptibility of VEXAS CD34^+^ cells to TAK‐243 presents a promising therapeutic approach for a more effective and less toxic treatment for this challenging disorder.

In summary, we here provide a strong rationale for further clinical investigation of TAK‐243 as a potential targeted therapy for patients with VEXAS syndrome. These findings offer hope for improving the management of this recently discovered disorder and contribute to our understanding of its underlying molecular mechanisms. Future studies should also explore combination therapies with standard MDS treatments for patients with concomitant myeloid neoplasms.

## AUTHOR CONTRIBUTIONS

N.S., D.N. and M.D. designed the study, analysed and interpreted data and wrote the manuscript. N.S., M.D. and A.K. performed experiments. A.S. executed bioinformatic analyses. V.R., E.A., F.R., A.‐C.B., T.K., M.H., F.H., V.N., N.W., J.O., I.P. and M.G. provided technical assistance. A.D. provided primary material from healthy controls. A.S., L.S., M.A. and G.M. provided patient material and clinical data. D.N. and W.‐K.H. supervised the whole study and provided research infrastructure.

## FUNDING INFORMATION

This study was supported by the ‘Forum Gesundheitsstandort Baden‐Württemberg’, BW 4‐5400/136/1 (D.N.); the German Cancer Aid Foundation, 70113953 (D.N.); the German Research Foundation, SFB 1709/1 2025—533056198 (D.N.); the Health + Life Science Alliance Heidelberg Mannheim (V.R.); state funds approved by the State Parliament of Baden‐Württemberg (V.R.); the ‘Bundesinstitut für Risikobewertung’, 60‐0102‐01.P638‐12710524 (E.A.); the ‘Dr. Werner Jackstädt‐Stiftung’ (E.A.); the Medical Faculty Mannheim of the Heidelberg University ‘SEED’ (N.S.) and the Torsten Haferlach Leukemia Diagnostics Foundation (N.S.).

## CONFLICT OF INTEREST STATEMENT

The authors declare no conflicts of interest.

## ETHICS STATEMENT

The study protocol was approved by the ethics committee of the Medical Faculty of Mannheim, Heidelberg University, and complies with the Declaration of Helsinki.

## PATIENT CONSENT STATEMENT

All primary patient samples used in this study were collected after obtaining written informed consent from the patients.

## Supporting information


Figure S1.

Figure S2.

Figure S3.

Figure S4.

Figure S5.

Tables S1–S3.

Appendix S1.

## Data Availability

The data supporting the findings of this study are available from the corresponding author upon reasonable request.
