# Supplementary figures and images for: Primary CD34+ cells of patients with vacuoles, E1 enzyme, X‐linked, autoinflammatory, somatic (VEXAS) syndrome are highly sensitive to targeted treatment with TAK‐243
Source: Br J Haematol. 2025 Oct 12;207(6):2558–62. doi: 10.1111/bjh.70203 (PMC12710189; doi:10.1111/bjh.70203)

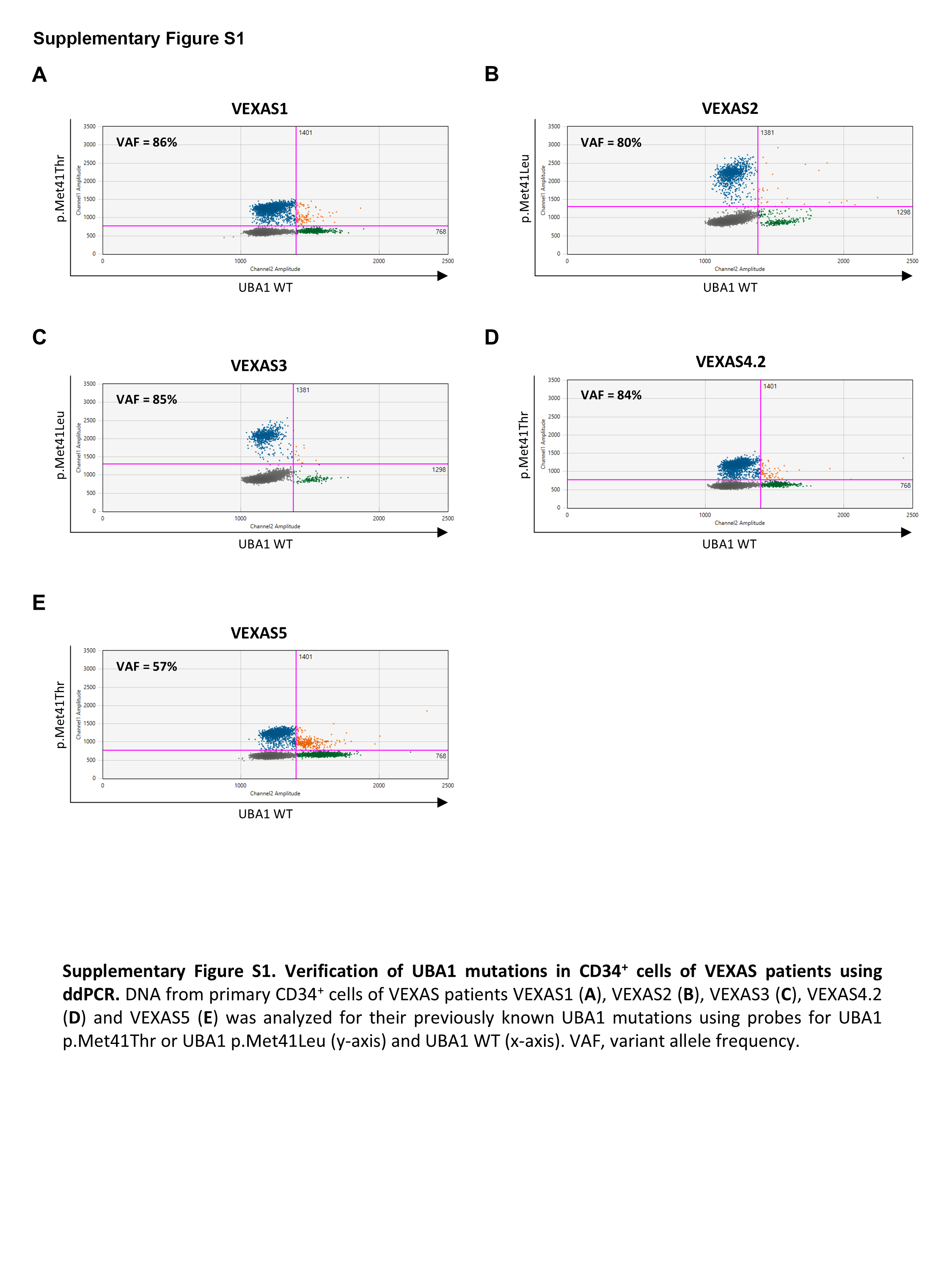

Supplement: Supplementary file 1 — Figure S1. [file BJH-207-2558-s006.tif]
