# Supplementary material for: Primary CD34+ cells of patients with vacuoles, E1 enzyme, X‐linked, autoinflammatory, somatic (VEXAS) syndrome are highly sensitive to targeted treatment with TAK‐243
Source: Br J Haematol. 2025 Oct 12;207(6):2558–62. doi: 10.1111/bjh.70203 (PMC12710189; doi:10.1111/bjh.70203)
Supplement: Supplementary file 6 — Tables S1–S3. [file BJH-207-2558-s001.docx]

**Supplementary Table S1. Detailed genetic and clinical characteristics of VEXAS patients.** CRP, C-reactive protein; ESA, erythropoiesis-stimulating agent; Hb, hemoglobin; IPSS-R/IPSS-M, Revised/Molecular International Prognostic Scoring System for MDS; MDS, myelodysplastic neoplasms; MDS-LB, MDS with low blasts (WHO 2022); PLT, platelets; WBC, white blood cells; VAF, variant allele frequency; *at time of bone marrow aspiration; **time from first diagnosis to death/last follow-up; *** 1) viability assay, 2) apoptosis assay, 3) VAF assay.

| **Patient ID** | **Sex** | **Age*** | **Overall survival**** | **Clinical manifestation*** | **Blood parameters*** | **Cytology*** | **Karyotype*** | **Mutations*** | **Treatment*** | **Used for assay***** |
| --- | --- | --- | --- | --- | --- | --- | --- | --- | --- | --- |
| **VEXAS1** | male | 74 | 14 months | - MDS-LB (IPSS-R: low, IPSS-M: low) - Fever - Papular and pustular rash on whole body - Petechiae on the lower legs | - Anemia (Hb: 9.2 g/dl) - Leukopenia (WBC: 4.17x10^9^/L) - Elevated CRP (82 mg/l) | - Vacuolization in the immature erythroid precursors - Dysplasia in erythropoiesis and granulopoiesis | 46, XY [10] | - UBA1 p.Met41Thr (VAF 89%) - DNMT3A (VAF 40%) | - Prednisolone | 1, 2, 3 |
| **VEXAS2** | male | 73 | 40 months | - MDS-LB (IPSS-R: low, IPSS-M: low) - Progressive cutaneous leukemic infiltrates (Sweet syndrome) | - Anemia (Hb: 9.3 g/dl) - Thrombocytopenia (PLT: 25x10^9^/L) - Elevated CRP (45.3 mg/l) | - Vacuolization in the erythroid and myeloid lineage - Dysplasia in erythropoiesis, granulopoiesis and megakaryopoiesis | 46, XY [20] | - UBA1 p.Met41Leu (VAF 82%) | - ESA - Asunercept - Iron chelator | 1 |
| **VEXAS3** | male | 74 | 10+ months | - MDS-LB (IPSS-R: intermediate, IPSS-M: moderate low) | - Anemia (Hb: 6.9 g/dl) - Leukopenia (WBC: 2.83x10^9^/L) - Thrombocytopenia (PLT: 72x10^9^/L) - Elevated CRP (61 mg/l) | - Vacuolization in promyelocytes - Dysplasia in megakaryopoiesis | 46,XY [20] | - UBA1 p.Met41Leu (VAF 83%) | - Prednisolone - Azathioprine | 1 |
| **VEXAS4.1**  **(= VEXAS4.2)** | male | 71 | 15+ months | - MDS-LB (IPSS-R: very low, IPSS-M: very low) - Fever of unknown origin - Arteritic anterior ischemic optic neuropathy - Maculopapular rash on the left upper arm - Mesenteric panniculitis - Recurrent polychondritis | - Anemia (Hb: 12.4 g/dl) - Leukopenia (WBC: 3.41x10^9^/L) - Elevated CRP (37 mg/l) | - Vacuolization in the precursors of granulopoiesis, predominantly in promyelocytes and proerythroblasts - Dysplasia in erythropoiesis, granulopoiesis and megakaryopoiesis | 46,XY [20] | - UBA1 p.Met41Thr (VAF 90%) | - Prednisolone | 1, 3 |
| **VEXAS4.2**  **(= VEXAS4.1)** | male | 72 | 15+ months | (see above) | - Anemia (Hb: 7.4 g/dl) - Elevated CRP (90.7 mg/l) | - Vacuolization in (pro)myelocytes - Dysplasia in granulopoiesis and megakaryopoiesis | 46,XY [20] | - UBA1 p.Met41Thr (VAF 87%) | - Prednisolone - Ruxolitinib | 2 |
| **VEXAS5** | male | 70 | 4+ months | - MDS-LB (IPSS-R: very low, IPSS-M: very low) - Recurrent polychondritis | - Elevated CRP (16.9 mg/l) | - Vacuolization in erythropoiesis and granulopoiesis - Dysplasia in erythropoiesis, granulopoiesis and megakaryopoiesis | 46,XY [20] | - UBA1 p.Met41Thr (VAF 36%) - SMC3 (VAF 16%) - DNMT3A (VAF 15%) | - Prednisolone | 2, 3 |

**Supplementary Table S2. Key clinical data of MDS patients and healthy controls.** ESA, erythropoiesis-stimulating agent; HY, healthy; IPSS-R/IPSS-M, Revised/Molecular International Prognostic Scoring System for MDS; int, intermediate; MDS, myelodysplastic neoplasms; MDS-LB, MDS with low blasts; MDS-SF3B1, MDS with SF3B1 mutation; WHO 2022, WHO classification of MDS 2022; *at time of bone marrow aspiration; ** 1) viability assay, 2) apoptosis assay, 3) VAF assay.

| **Patient ID** | **Sex** | **Age*** | **Diagnosis (WHO 2022)*** | **IPSS-R*** | **IPSS-M*** | **Karyotype*** | **Mutations*** | **Therapy*** | **Used for assay**** |
| --- | --- | --- | --- | --- | --- | --- | --- | --- | --- |
| **MDS1** | female | 60 | MDS-LB | low | low | 46,XX [20] | TET (13%), STAG2 (11%) | none | 1 |
| **MDS2** | female | 75 | MDS-LB | int | moderate high | 46,XX [20] | 3x TET2 (35%/37%/37%), ASXL1 (30%), 2x EZH2 (29%/34%) | Iron chelator | 1 |
| **MDS3** | male | 79 | MDS-LB | int | moderate low | 46,XY [20] | U2AF1 (29%), DNMT3A (8%) | none | 1 |
| **MDS4** | female | 70 | MDS-SF3B1 | very low | very low | 46,XX [20] | SF3B1 (40%) | none | 1 |
| **MDS5** | male | 41 | MDS-SF3B1 | low | low | 46,XY [20] | SF3B1 (43%), NRAS (18%), TET2 (10%) | none | 1 |
| **MDS6** | male | 76 | MDS-SF3B1 | low | low | 46,XY [26] | SF3B1 (37%), DNMT3A (35%) | ESA | 2 |
| **MDS7** | female | 65 | MDS-LB | very low | low | 46,XX [20] | DNMT3A (5%) | none | 2 |
| **MDS8** | female | 84 | MDS-LB | low | high | 46,XX [20] | IDH2 (38%), ASXL1 (16%), SRSF2 (13%), PHF6 (3%) | none | 2 |
| **MDS9** | male | 74 | MDS-LB | low | moderate low | 46,XY [20] | ASXL1 (23%), 2x IDH1 (22%/8%), SRSF2 (21%), NF1 (9%), NRAS (5%) | Prednisolone | 2 |
| **MDS10** | female | 46 | MDS-SF3B1 | low | very low | 46,XX [20] | SF3B1 (38%), CUX1 (2%) | none | 2 |
| **HY1** | female | 60 | hematologically healthy | - | - | - | - | - | 1 |
| **HY2** | male | 53 | hematologically healthy | - | - | - | - | - | 1 |
| **HY3** | female | 63 | hematologically healthy | - | - | - | - | - | 1 |
| **HY4** | female | 84 | hematologically healthy | - | - | - | - | - | 1 |
| **HY5** | male | 76 | hematologically healthy | - | - | - | - | - | 1 |
| **HY6** | female | 87 | hematologically healthy | - | - | - | - | - | 2 |
| **HY7** | male | 58 | hematologically healthy | - | - | - | - | - | 2 |
| **HY8** | male | 53 | hematologically healthy | - | - | - | - | - | 2 |
| **HY9** | male | 43 | hematologically healthy | - | - | - | - | - | 2 |
| **HY10** | female | 65 | hematologically healthy | - | - | - | - | - | 2 |

**Supplementary Table S3. UBA1 VAF of VEXAS CD34^+^ cells after treatment with TAK-243 and pevonedistat.** Non-expanded and expanded CD34^+^ cells of patients VEXAS1, VEXAS4 and VEXAS5 were treated with the IC25, IC50 and IC75 values od TAK-243 or pevonedistat for 48 hours. The cells were then harvested for DNA isolation. The variant allele frequency (VAF) of UBA1 for each condition was determined by ddPCR. Vehicle control was set to 100% and the other values were normalized accordingly. The results normalized for vehicle control are shown in **Figure 2A**+**B**.

| **UBA1 VAF (%)** | **VEXAS1**  **(expanded CD34^+^ cells)** | **VEXAS4**  **(expanded CD34^+^ cells)** | **VEXAS5**  **(non-expanded CD34^+^ cells)** |
| --- | --- | --- | --- |
| **Vehicle** | 44.0 | 15.5 | 34.8 |
| **TAK-243 IC25** | 21.6 | 9.1 | 33.7 |
| **TAK-243 IC50** | 19.1 | 9.4 | 23.5 |
| **TAK-243 IC75** | 22.4 | 5.0 | 16.7 |
| **Pevonedistat IC25** | 29.2 | 11.3 | 24.0 |
| **Pevonedistat IC50** | 45.0 | 18.3 | 24.0 |
| **Pevonedistat IC75** | 37.5 | 26.0 | 30.0 |
