# Supplementary material for: Primary CD34+ cells of patients with vacuoles, E1 enzyme, X‐linked, autoinflammatory, somatic (VEXAS) syndrome are highly sensitive to targeted treatment with TAK‐243
Source: Br J Haematol. 2025 Oct 12;207(6):2558–62. doi: 10.1111/bjh.70203 (PMC12710189; doi:10.1111/bjh.70203)
Supplement: Supplementary file 7 — Appendix S1. [file BJH-207-2558-s003.docx]

**Supplementary Methods**

**Primary samples**

Primary bone marrow (BM) samples from patients with VEXAS (n=5) or MDS (n=10) were obtained from residual diagnostic BM aspirations. BM of healthy, age-matched controls (n=10) was isolated from femoral heads received after hip replacement surgery. All samples were collected after obtaining the patients’ written informed consent and in accordance with the Institutional Review Board statement of the Medical Faculty Mannheim and the Declaration of Helsinki. Detailed information on patients and controls is provided in **Supplementary Tables S1**+**2**. BM-MNCs were isolated by red cell lysis. Enrichment of CD34^+^ cells from MNCs by magnetic cell separation was performed using the “CD34 MicroBead” kit (Miltenyi Biotech) yielding a purity of at least 90%. Enriched CD34^+^ cells were viably frozen until further use.

**Droplet digital PCR**

DNA from CD34^+^ cells was isolated using the “AllPrep DNA/RNA” kit (QIAGEN). The determination of the UBA1 variant allele frequency (VAF) by droplet digital PCR was performed using the “QX200 Droplet Digital PCR System” (Bio-Rad Laboratories). Primers (forward: 5’-CTCCACTCCTGTGTGTCT-3’, reverse: 5’-GTAAAGGCCCTCGTCTATGT-3’) and probes for UBA1 wild type (5’-HEX-CTAGGGAATGGC-3’), UBA1 p.Met41Thr (5’-FAM–CTAGGGAACGGC-3’) and UBA1 p.Met41Leu (5’-FAM–CTAGGGACTGGC-3’) were purchased as ready-to-use mix in a ratio of 900:575 nM (Bio-Rad Laboratories). Droplets were generated using the “QX200 Droplet Generator” (Bio-Rad Laboratories). PCR reactions were performed using the “C1000 Touch Thermal Cycler with 96–Deep Well Reaction Module” (Bio-Rad Laboratories). Droplets were counted using the “QX200 Droplet Reader” (Bio-Rad Laboratories). Analysis was performed using the “QX Manager” software (Bio-Rad Laboratories, v2.0.0).

**Cell viability and apoptosis assays**

After thawing, CD34^+^ cells were cultured in “StemSpan SFEM II” medium (STEMCELL Technologies) plus cytokines (10 ng/ml FGF-1, 50 ng/ml FLT3-L, 50 ng/ml SCF, 10 ng/ml TPO) and 1% pen-strep overnight. To set up the assays, 2,500 CD34^+^ cells per well in StemSpan™ SFEM II plus cytokines in replicates of 3-5 were treated with different concentrations of TAK-243 (range: 0.24–1,000 nM) and pevonedistat (range: 0.78–3,000 nM) or vehicle (DMSO) for 48 hours. Cell viability and apoptosis were then assessed using the “CellTiter-Glo Luminescent Cell Viability Assay” or “Caspase-Glo 3/7 Assay” (Promega), respectively. The luminescence was measured using the “Tecan Infinite 200 PRO 8” microplate reader (Tecan Group Ltd.). All results were normalized according to the DMSO results. The raw data of the viability assay results not normalized to the DMSO control can be found in the **Supplementary Figures S5A**-**F**.

**CD34^+^ cell expansion**

To determine the UBA1 VAF in the VEXAS CD34^+^ cells after treatment with TAK-243 and pevonedistat, n=2 samples had to be expanded beforehand to obtain enough cells for DNA isolation. For this purpose, the CD34^+^ cells were expanded in StemSpan™ SFEM II and “StemSpan Myeloid Expansion Supplement” (STEMCELL Technologies) for 7–10 days until the cell count was sufficient. The expanded CD34+ cells were then harvested and treated with TAK-243 and pevonedistat (IC25, IC50, IC75). After 48 hours, the cells were harvested again and used for DNA isolation.

**Statistics**

Statistical analysis was performed using Prism 10.5.0 (GraphPad Software). Data were analyzed using nonlinear regression, two-tailed unpaired t-test and ordinary one-way or two-way ANOVA. Error bars are represented as mean ± SD. P values less than 0.05 were considered significant.
